# Development and evaluation of inactivated vaccines incorporating a novel Senecavirus A strain-based Immunogen and various adjuvants in mice

**DOI:** 10.3389/fvets.2024.1376678

**Published:** 2024-05-03

**Authors:** Bingliang Wang, Fei Gao, Ruijie Hu, Hanrong Huyan, Gaili Wang, Zezhao Cao, Yue Zhao, Huijun Lu, Deguang Song, Feng Gao, Wenqi He, Yungang Lan

**Affiliations:** ^1^State Key Laboratory for Diagnosis and Treatment of Severe Zoonotic Infectious Diseases, Key Laboratory for Zoonosis Research of the Ministry of Education, Institute of Zoonosis, and College of Veterinary Medicine, Jilin University, Changchun, China; ^2^Department of Laboratory Animals, Jilin Provincial Key Laboratory of Animal Model, Jilin University, Changchun, China; ^3^Jilin Province Animal Disease Control Center, Changchun, China; ^4^Jilin Academy of Animal Husbandry and Veterinary Medicine, Changchun, China

**Keywords:** Senecavirus A (SVA), inactivated vaccine, MONTANIDETM GEL02 PR (GEL 02), immune response, adjuvants

## Abstract

Porcine idiopathic vesicular disease (PIVD), one of several clinically indistinguishable vesicular diseases of pigs, is caused by the emerging pathogen Senecavirus A (SVA). Despite the widespread prevalence of porcine SVA infection, no effective commercial vaccines for PIVD prevention and control are available, due to high costs associated with vaccine testing in pigs, considerable SVA diversity, and SVA rapid evolution. In this study, SVA CH/JL/2022 (OP562896), a novel mutant SVA strain derived from an isolate obtained from a pig farm in Jilin Province, China, was inactivated then combined with four adjuvants, MONTANIDETM GEL02 PR (GEL 02), MONTANIDETM ISA 201 VG (ISA 201), MONTANIDETM IMG 1313 VG N (IMS1313), or Rehydragel LV (LV). The resulting inactivated SVA CH/JL/2022 vaccines were assessed for efficacy in mice and found to induce robust *in vivo* lymphocyte proliferation responses and strong IgG1, IgG2a, and neutralizing antibody responses with IgG2a/IgG1 ratios of <1. Furthermore, all vaccinated groups exhibited significantly higher levels of serum cytokines IL-2, IL-4, IL-6, and IFN as compared to unvaccinated mice. These results indicate that all vaccines elicited both Th1 and Th2 responses, with Th2 responses predominating. Moreover, vaccinated mice exhibited enhanced resistance to SVA infection, as evidenced by reduced viral RNA levels and SVA infection-induced histopathological changes. Collectively, our results demonstrate that the SVA-GEL vaccine induced more robust immunological responses in mice than did the other three vaccines, thus highlighting the potential of SVA-GEL to serve an effective tool for preventing and controlling SVA infection.

## 1 Introduction

Senecavirus A (SVA), formally known as Seneca Valley virus (SVV), is a causative agent of porcine idiopathic vesicular disease (PIVD) (1, 2). SVA is a non-enveloped, single-stranded, positive-sense RNA virus belonging to the genus *Senecavirus* within the family *Picornaviridae* (2). The SVA genome is approximately 7.3 kb in length and consists of a 5′-untranslated region (UTR), a 3’-UTR, and a single open reading frame (ORF) (2). This ORF encodes a polyprotein that is subsequently cleaved to form twelve mature proteins. The polyprotein is comprised of a 5′ leader peptide (L), four structural proteins (VP1, VP2, VP3, VP4), three non-structural proteins (2A, 2B, 2C), and four other non-structural proteins (3A, 3B, 3C, 3D) (2). These elements are arranged from the N-terminal end to the C-terminal end in the standard order ‘L-4-3-4,’ a characteristic feature of picornavirus polyproteins (2).

Shortly after the discovery of SVA, the pig-farming industry failed to perceive SVA as a significant threat, due to its low infection rate in pigs and apparent lack of pathogenicity. However, in 2007 a Canadian pig farm reported SVA-induced PIVD cases characterized by clinical signs resembling idiopathic vesicular disease (IVD) (1). These signs included broken vesicles running along the coronary band junction between the hoof and leg resulting in coronary band swelling and blanching, tissue separation between hoof pads and edges, and dewclaw sloughing from legs. In addition, the previous studies have shown that SVA nucleic acids was detected in mice, houseffies and culicoides, indicating them may also be the natural hosts (3, 4).

Since the end of 2014, large SVA outbreaks have occurred in swine herds in various regions of North America, Brazil, and other countries (5–8). Following these outbreaks, the first reported outbreak in China took place in 2015 and subsequently spread to Hubei, Henan, Heilongjiang, Fujian, and other provinces, resulting in markedly reduced pork production and substantial economic losses (8–12). Intriguingly, as the virus continued to spread in China, the initial high SVA virulence towards farmed pigs gradually diminished (13). This change led to frequent subclinical infections, making it challenging to detect infected pigs early and control disease transmission effectively. As a result, the emergence and spread of novel SVA subtypes have posed significant challenges to the global pork industry. This threat has been exacerbated by the absence of commercially available effective vaccines for preventing and controlling SVA infection and transmission. So far, inactivated vaccines, live attenuated vaccines, viruslike particle vaccines, and subunit vaccines of SVA have been developed in previous studies (8, 14, 15). Among them, inactivated vaccines are traditional and widely used vaccines that work by producing a non-toxic vaccine that can prevent infection.

The development of effective vaccine candidates to prevent SVA infection and transmission faces several challenges, such as high costs associated with vaccine safety and performance testing in pigs, SVA rapid evolution, and the lack of suitable SVA-susceptible animal models. Nevertheless, the use of mouse models for evaluating SVA vaccine candidates have provided valuable insights into factors associated with vaccine efficacy (3, 8). In addition, some studies underscore the critical role of adjuvant formulations in enhancing the SVA-inactivated vaccine-induced adaptive immune responses (16, 17). However, the more appropriate adjuvant for preparing SVA-inactivated vaccine is still needed to be further screened.

In this study, we prepared four inactivated vaccines by combining an immunogen, a novel SVA strain CH/JL/2022 isolated in Jilin Province, with four different adjuvants: MONTANIDETM GEL02 PR (GEL 02), MONTANIDETM ISA 201 VG (ISA 201), MONTANIDETM IMG 1313 VG N (IMS1313), and Rehydragel LV (LV). These candidate vaccines, designated SVA-ISA 201 (SVA-ISA), SVA-IMS1313 (SVA-IMS), SVA-GEL 02 (SVA-GEL), and SVA-LV, were administered to mice then vaccine immunogenicity, and *in vivo* protection against SVA infection were evaluated to enable the selection of the most suitable adjuvant for incorporation in an inactivated SVA vaccine.

## 2 Materials and methods

### 2.1 Cells, clinical samples, animals and ethics statement

Baby Hamster Syrian Kidney (BHK-21 cells) were cultured in an improved basic culture medium containing (Dalian, China) 6% fetal bovine serum (GIBCO) with 1% penicillin–streptomycin solution at 37°C in a 5% CO2 constant temperature incubator. Lymphoid tissue samples of diseased pigs were collected from a pig farm in Jilin Province, China, where the diseased pigs had typical clinical symptoms of vesicular disease. Three-week-old male BALB/c mice with masses between 13 and 15 g were purchased from Liaoning Changsheng Biotechnology Co., Ltd., China.

### 2.2 Virus isolation, full-length genome sequencing and titrations

Virus isolation was performed in a BHK-21 cell culture system from SVA-positive vesicular fluid samples as described previously (8). In brief, Monolayers of BHK-21 cells were inoculated with the simples diluted with serum-free DMEM and filtrated through 0.22 μm filters. The virus strains were collected at 72 h post-infection (hpi) with the appearance of obvious cytopathic effects (CPE), and subjected to three freeze–thaw cycles. The supernatant of lysate was collected after 2000 rpm for 20 min at 4°C and further confirmed by PCR analysis with SVA-specific primers and the sequences were as follows: SVA-F, 5′- CACACTGCCAACGTCCCTTA-3′; SVA-R, 5′- CCAAGGAGAAAGCCAGGATG-3′. Genome sequencing of the SVA isolate was performed by Sangon Biotech Co., Ltd. (Shanghai, China) with an Illumina HiSeq. 2,500 system, and the genomic sequences of which was deposited in GenBank,^1^ and named as SVA CH/JL/2022. *The virus solution purified* by sucrose density gradient centrifugation *after low-speed centrifugation at 40,000 rpm for 4 h at 4°C, was examined with transmission electron microscopy (TEM)* as described previously (12, 18). *In addition,* the viral titer was determined from BHK-21 cells using the median tissue culture infective dose (TCID_50_) and plaque assay as described previously (8, 12, 19). For TCID_50_ assay, CPE in BHK-21 cells were monitored for 72 h, and the viral titers were calculated according to the Spearman-Karber method (12). For plaque assay, BHK-21 cells in 6-well plates were infected with SVA CH/JL/2022 for 72 h in DMEM containing 2% low-melting-point agarose (Sigma-Aldrich) and 2% heat-inactivated Fetal Bovine Serum. For visualization, cells fixed in 4% paraformaldehyde, were stained with 0.5% crystal violet and observed after washing the plate with PBS (19).

### 2.3 SVA complete genome sequence analysis

Full-length nucleotide and amino acid sequence alignments between SVA CH/JL/2022 and other 22 SVV strains published from China and other countries were performed by MegAlign software using MUSCLE.

### 2.4 Preparation of inactivated vaccines and animal experiment

Formaldehyde at final concentrations of 1‰, 2‰, 3‰, 4‰ and 5‰ were used for virus inactivation, respectively. Different concentrations of formaldehyde were incubated with SVA at 37°C and the mixture was collected after 48 h, and then monolayers of BHK-21 cells were inoculated with the simples, respectively. The cell-virus mixture was collected for PCR detection after third blind passages. The inactivated viral antigen (SVA CH/JL/2022, virus titer of 10^6.375^ TCID_50_/mL) was emulsified with the adjuvants MONTANIDETM GEL02 PR (GEL 02), MONTANIDETM ISA 201 VG (ISA 201), MONTANIDETM IMG 1313 VG N (IMS1313), and Rehydragel LV (LV) at a volume ratio of 1:1, respectively. The prepared inactivated vaccines were used for mice immunization experiment.

Three-week-old male BALB/c mice were selected and randomly divided into 5 groups: PBS control group, SVA-ISA immunization group, SVA-IMS immunization group, SVA-GEL immunization group and SVA-LV immunization group, with 10 mice in each group. Mice from the individual groups were injected with 0.3 mL (1 dose) of the corresponding vaccine. Three mice were randomly selected from each group after the initial immunization by multipoint subcutaneous injection, and the changes in their body weights, body temperatures, and mental status were monitored for 10 days of consecutive. The remaining mice (*n* = 7) were injected with a first-boost after 2 weeks. Serum was collected at 0, 7, 14, 21, and 28 d after immunization and stored at −80°C for use.

### 2.5 SVA specific IgG, mouse IgG1 and IgG2a, and cytokine assays detection by ELISA

As previously described, SVA-VP3-specific IgG from immunized mice were detected by ELISA (20). Briefly, ELISA plates were coated with 500 ng/well of SVA-VP3 protein developed in our laboratory and samples were examined for anti-SVA antibody reactivity. In addition, Mouse IgG1 and IgG2a antibody levels in mouse serum were measured by using ELISA kits (Jiang Lai, Shanghai, China), respectively. In addition, mouse interleukin 2 (IL-2), mouse interleukin 4 (IL-4), mouse interleukin 6 (IL-6), and mouse interferon (IFN) ELISA kits (Jiang Lai, Shanghai, China) were used to investigate the change in cytokine levels of the mouse serum according to the instructions, respectively.

### 2.6 Virus neutralization (VN) and lymphocyte proliferation assay

As previously described, VN test was performed on the SVA CH/JL/2022 strain to determine the levels of neutralizing antibodies in the mice serum at 7 d after the mice received booster immunizations (21). In brief, the mice serum was heated at 56°C for 30 min and then 100 μL two-fold serial dilutions serum was co-incubated with 100 μL of 100 TCID_50_ SVA at 37°C for 1 h. The virus-serum mixture was transferred to 96-well plates of BHK-21 cells, which were further evaluated by CPE assay and the neutralizing antibodies levels were canulated by the Reed-Muench method.

On the 28th day after the initial immunization, spleens were randomly collected from 3 mice in each group, and lymphocytes of spleens were isolated as described previously (22, 23). The isolated lymphocytes were re-suspended in DMEM and cultured in 96-well flat bottom plates, and stimulated with concanavalin A (ConA, Sigma) at a final concentration of 5 μg/mL or 50 μL of inactivated virus (10^6.375^ TCID_50_/0.1 mL), respectively. DMEM simple was used as a negative control. Experiments were repeated three times. After 48 h, 10 μL/well of CCK-8 was added on the plates and further incubated for 4 h. The stimulation index (SI) was calculated as the value of (OD sample well-OD blank well) / (OD negative well-OD blank well) based on the OD 450 value measured at 450 nm.

### 2.7 Challenge and samples collection

On the 14th day after the booster immunization, the mice were inoculated with 300 μL SVA (10^6.375^ TCID_50_/ mL) via intraperitoneal (IP). Subsequently, SVA-infected mice at 14 days post-infection were sacrificed by CO2 asphyxiation according to animal handling guidelines. After sacrifice, the hearts, livers, spleens, lungs, kidneys, and duodenum of each mouse was surgically excised and placed on an ice pad for further testing.

### 2.8 Viral extraction and quantitative real-time PCR (qRT-PCR)

The total RNA of fresh tissues was extracted by Trizol Reagent (Invitrogen, United States). Reverse transcription (RT) was followed by using the reverse transcription kit (Vazyme, China). As previously reported, the SVA distributions in mice were determined by SYBR Green I real-time RT-PCR assay with the specific primers and the sequences were as follows: SVA-F, 5′-AGAATTTGGAAGCCATGCTCT-3′; SVA-R,5′-GAGCCAACATAGARACAGATTGC -3′ (8). The results were expressed as log_10_ RNA copies/mg. The data represent results from one representative triplicate experiment.

### 2.9 Histopathological examination (HE) and calcium deposit analysis

HE of mice tissues were performed to assess the lesions as described previously (24). In brief, the collected tissues were fixed in 10% formalin solution for 48 h at room temperature, dehydrated with different ethanol concentrations, and fixed in paraffin and sectioned, and HE staining was performed on thin sections (3–4 μm thickness). For calcium deposit analysis, 7 μm transverse sections of tissues were stained with the Von Kossa Stain Kit (Calcium Stain) (Abcam), following the manufacturer’s instructions and the sections were observed under a microscope.

### 2.10 Statistical analysis

The statistical tests were performed using GraphPad Prism v9.0 software (GraphPad Software, San Diego, CA). Data are presented as the means ± SD. Statistical analyses were performed on data from triplicate experiments by using Student’s t-test. In all cases, a *p* value of ≤0.05 was considered statistically significant.

## 3 Results

### 3.1 SVA isolation and characterization

**Figure 1:**
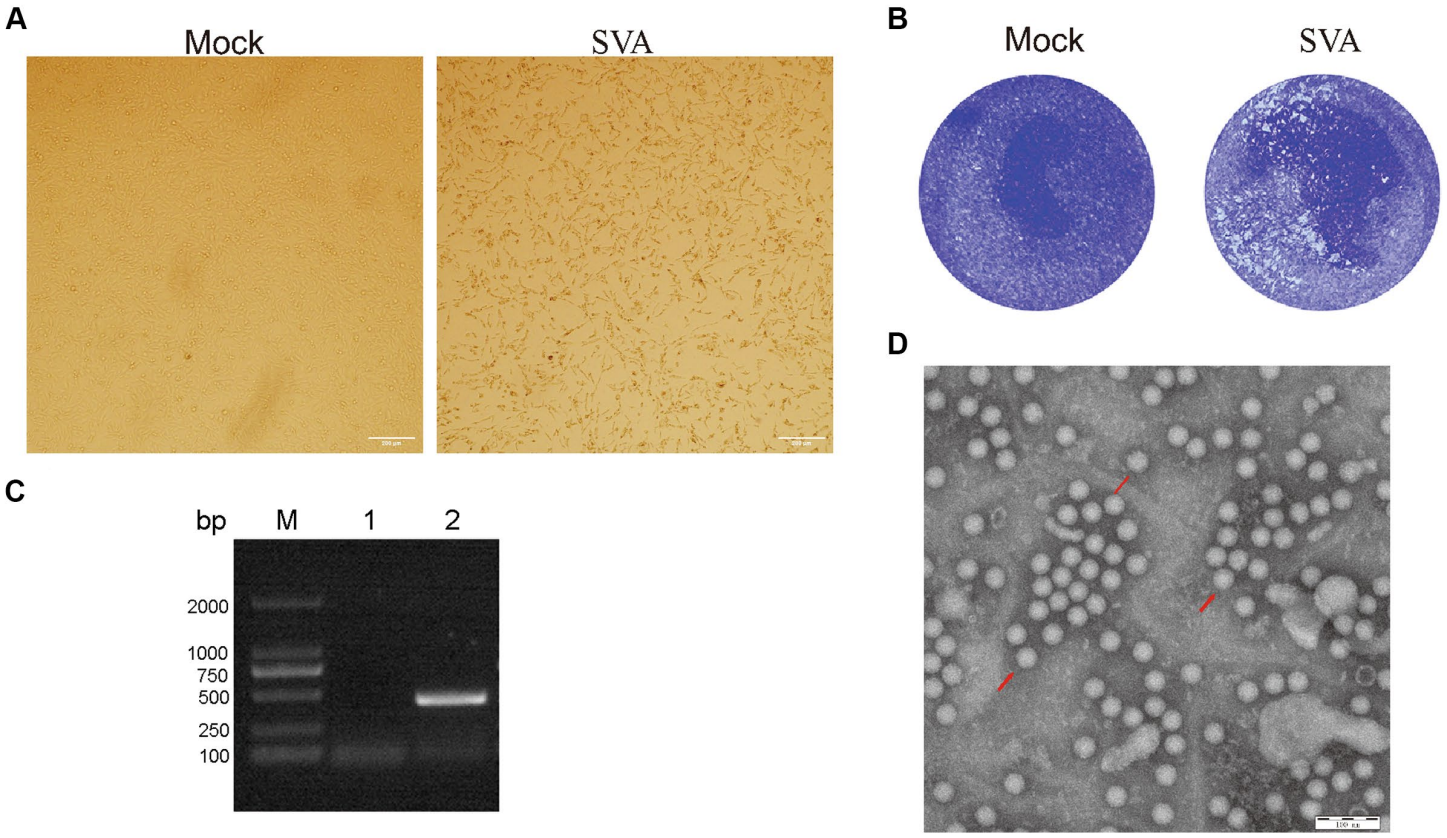
Isolation and characterization of the SVA CH/JL/2022 strain. **(A)** Typical cytopathic effects observed for BHK-21 cells after infection with the SVA CH/JL/2022 strain for 72 h. **(B)** Plaque assay of BHK-21 cells infected with the SVA CH/JL/2022 strain. **(C)** Results of PCR amplification of RNA isolated from culture supernatants of BHK-21 cells infected with fourth-generation SVA CH/JL/2022 virus. M: BM2000+ DNA Marker; 1: Negative control; 2: Amplified SVA-specific fragments. **(D)** Electron microscopy image showing viral particles of the SVA CH/JL/2022 strain.

The lymph nodes positive for SVA by RT-PCR (data not shown), collected in 2022 from SVA-infected pigs residing on a farm in Jilin Province, China, were stored in our laboratory. Samples containing SVA were processed and separately inoculated onto monolayers of BHK-21 cells. The fourth-generation virus was found to induce a stable cytopathic effect (CPE) when cultured with BHK-2 cells for 72 h; CPE characteristics included noticeable cell shrinkage, cell detachment from flask surfaces, and a lack of cell–cell adhesion (Figure 1A). Plaque assays were performed by inoculating BHK-21 cells with the SVA CH/JL/2022 strain, followed by cell staining with crystal violet. These assays revealed that this SVA strain induced noticeable plaque formation when added to BHK-21 cell monolayers (Figure 1B).

To determine whether SVA was present in cell culture supernatants of SVA-infected BHK-21 cells, we conducted PCR amplification-based detection of viral sequences in RNA extracted from culture supernatants via repeated freeze–thaw cycles. The results of these assays yielded positive SVA-detection results (Figure 1C). Whole-genome sequencing of cDNA prepared from an SVA-positive supernatant confirmed that the passaged virus represented a novel SVA strain, which was designated SVA CH/JL/2022.

To confirm that SVA particles were released from infected BHK-21 cells, the culture supernatant of infected cells was concentrated through ultracentrifugation then was subjected to negative staining. After the field of view was adjusted, examination of the stained concentrated supernatant under an electron microscope clearly revealed the presence of viral particles with diameters of 25 nm (Figure 1D). The titer of fourth-generation progeny virus in the culture supernatant of SVA-infected BHK-21 cells was calculated using the Spearman-Karber method and found to approach 10^6.375^ TCID_50_/ml. These experimental results confirm the successful isolation of SVA and establish that SVA CH/JL/2022 can effectively infect BHK-21 cells and that SVA can be propagated using that cell line, thereby laying a solid foundation for SVA vaccine production.

### 3.2 Phylogenetic tree construction and sequence analysis

**Figure 2:**
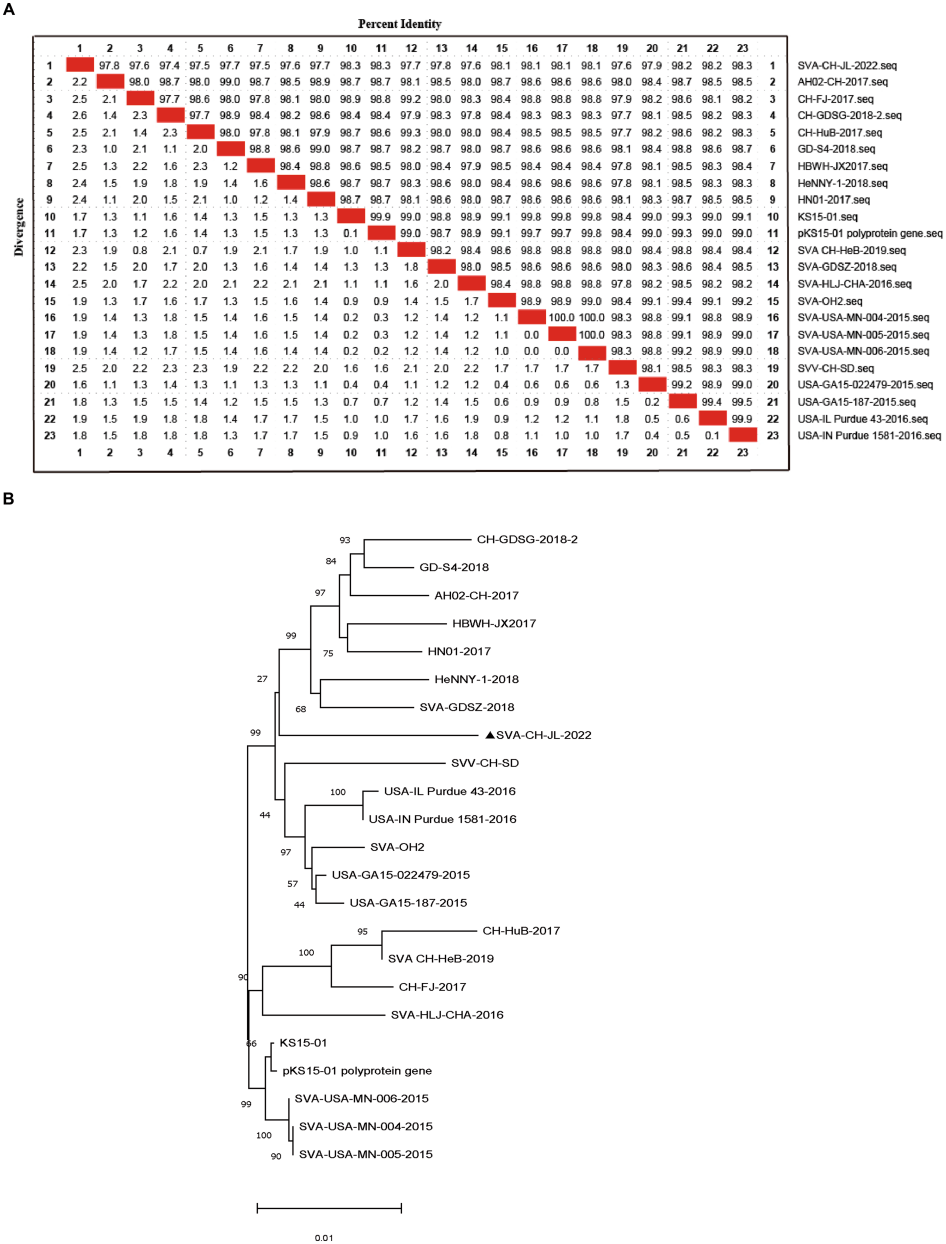
Sequence analysis of SVA CH/JL/2022 genomic DNA. **(A)** Plot of nucleotide sequence homology analysis results obtained for the SVA genome. **(B)** Phylogenetic tree based on results of whole-genome sequencing of the SVA CH/JL/2022 strain and genome sequences of 22 reference SVA strains.

Based on whole-genome sequencing results, the size of the SVA CH/JL/2022 genome was found to be 7,278 bp and a single coding sequence (CDS) region extending from 638 bp to 7,183 bp was present in the genome. The complete SVA CH/JL/2022 genome sequence and associated relevant details were submitted to the GenBank database under the accession number OP562896. We also obtained genomic sequences of 22 other SVA strains from GenBank to serve as reference sequences. Using MegAlign, nucleotide sequence homology rates between genomic sequences of SVA CH/JL/2022 and reference strains were determined. The results revealed nucleotide sequence homology rates ranging from 97.4 to 98.3% between the novel SVA CH/JL/2022 strain and the reference strains. The lowest and highest nucleotide sequence homology rates of 97.4 and 98.3% were obtained through comparisons between the SVA CH/JL/2022 sequence and sequences of the Chinese CH-GDSG-2018-2 isolate and the foreign isolate KS15-01, USA-IN Purdue 1,581–2016, respectively (Figure 2A).

MEGA 11.0.13 was used to construct a phylogenetic tree based on sequences obtained for the SVA CH/JL/2022 strain and other reference strains (Figure 2B). Notably, the novel SVA CH/JL/2022 isolate characterized in this study was evolutionarily more closely related to SVA-GD-SZ-2018 and SVA-CH-HeB-2019, and formed an evolutionary branch.

Comparisons of the SVA CH/JL/2022 polyprotein amino acid sequence with corresponding sequences from representative SVA reference strains revealed unique amino acid substitutions in the SVA CH/JL/2022 polyprotein that included P411S, R492Q, V493M, E582K, Q1172H, T1474A, I1553S, and K1864R (Table 1). Moreover, multiple amino acid differences were found between the SVA CH/JL/2022 polyprotein sequence and that of the SVV-001 strain, such as K56R, I165T, S368N, F426Y, T428A, I452L, G491E, S494P, E497V, A502T, A511T, K516R, A575T, I603V, A735Q, T736E, V766A, D770G, Y834F, T845A I894V, V912I, N988D, I1003V, T1079K, G1148S, S1366N, A1378T, T1415S, A1427T, D1429E, K1462R, T1469A, E1478G, P1479S, A1480V, A1547T, L1589I, A1628S, D1649E, M1685L, V1729I, S1839N, A1850V, P1856A, and M1860V (Table 1).

**Table 1:** Amino-acid differences in the major genes of different strains of SVA.

Virus	Protein positions (AA)																																																					
	56	165	368	426	428	441	452	491	492	493	494	497	502	511	516	575	582	603	735	736	766	770	834	845	894	912	988	1,003	1,079	1,148	1,172	1,366	1,378	1,415	1,427	1,429	1,462	1,469	1,474	1,478	1,479	1,480	1,547	1,553	1,589	1,628	1,649	1,685	1729	1839	1850	1856	1860	1864
SVA-CH-JL-2022						S			Q	M							K														H								A					S										R
SVV-001	R	T	N	Y	A		L	E			P	V	T	T	R	T		V	Q	E	A	G	F	A	V	I	D	V	K	S		N	T	S	T	E	R	A		G	S	V	T		I	S	E	L	I	N	V	A	V	
AH02-CH-2017																																																						
CH-FJ-2017																																																						
HBWH-JX2017	R																																																					
KS15-01																																																						
SVA-GDSZ-2018																	K					K																																
SVA-USA-MN-004-2015																																			V																			
USA-GA15-187-2015																																											T					L						
USA-IL_Purdue_43–2016																																			T													L						
Majority	K	I	S	F	T	P	I	G	R	V	S	E	A	A	K	A	E	I	A	T	V	D	Y	T	I	V	N	I	T	G	Q	S	A	T	A	D	K	T	T	E	P	A	A	I	L	A	D	M	V	S	A	P	M	K

The abovementioned findings suggest that the novel isolate SVA CH/JL/2022 may be closely related to the Chinese SVA strain. This result underscores the need for further investigations of the impact of SVA polyprotein amino acid substitutions on viral CPE and immunogenicity; such studies are urgently needed to guide the development of strategies to prevent and control SVA infection and transmission in the face of continuous SVA mutation and genomic recombination.

### 3.3 Virus inactivation

**Figure 3:**
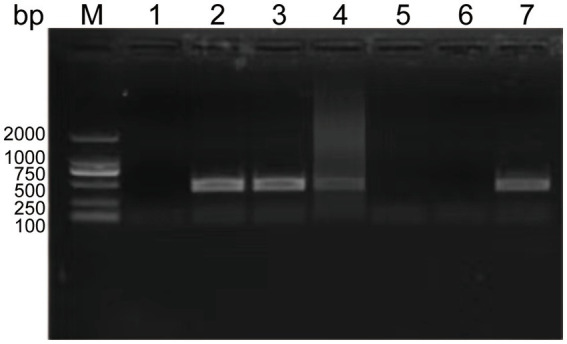
Experimental results showing effects of various formaldehyde concentrations on SVA inactivation and vaccine safety in mice. PCR-based detection results for culture supernatants containing SVA CH/JL/2022 after the virus was blindly passaged for three generations then inactivated. M: BM2000+ DNA Marker; 1: Negative control; 2–6: PCR-based detection results for culture supernatants containing SVA CH/JL/2022 after the virus was blindly passaged for three generations then inactivated in various final formaldehyde concentrations within the range of 1‰-5‰; 7: Positive control.

To determine the minimal formaldehyde concentration required for complete inactivation of the SVA CH/JL/2022 strain, we treated samples of this strain with formaldehyde at final concentrations ranging from 1‰ to 5‰. After treatment, both formaldehyde-treated and untreated SVA samples were added to BHK-21 cell cultures and blindly passaged for 3 generations. Culture supernatants were then collected and RNA was extracted for use in PCR-based detection of SVA RNA. The results indicated that culture supernatants of cells exposed to formaldehyde at final concentrations of 4‰ and 5‰ tested negative for SVA, thus demonstrating effective SVA inactivation. Therefore, we adopted the lowest effective formaldehyde concentration of 4‰ for SVA inactivation in this study (Figure 3).

### 3.4 Study of specific antibodies induced in mice immunized with inactivated vaccines containing different adjuvants

**Figure 4:**
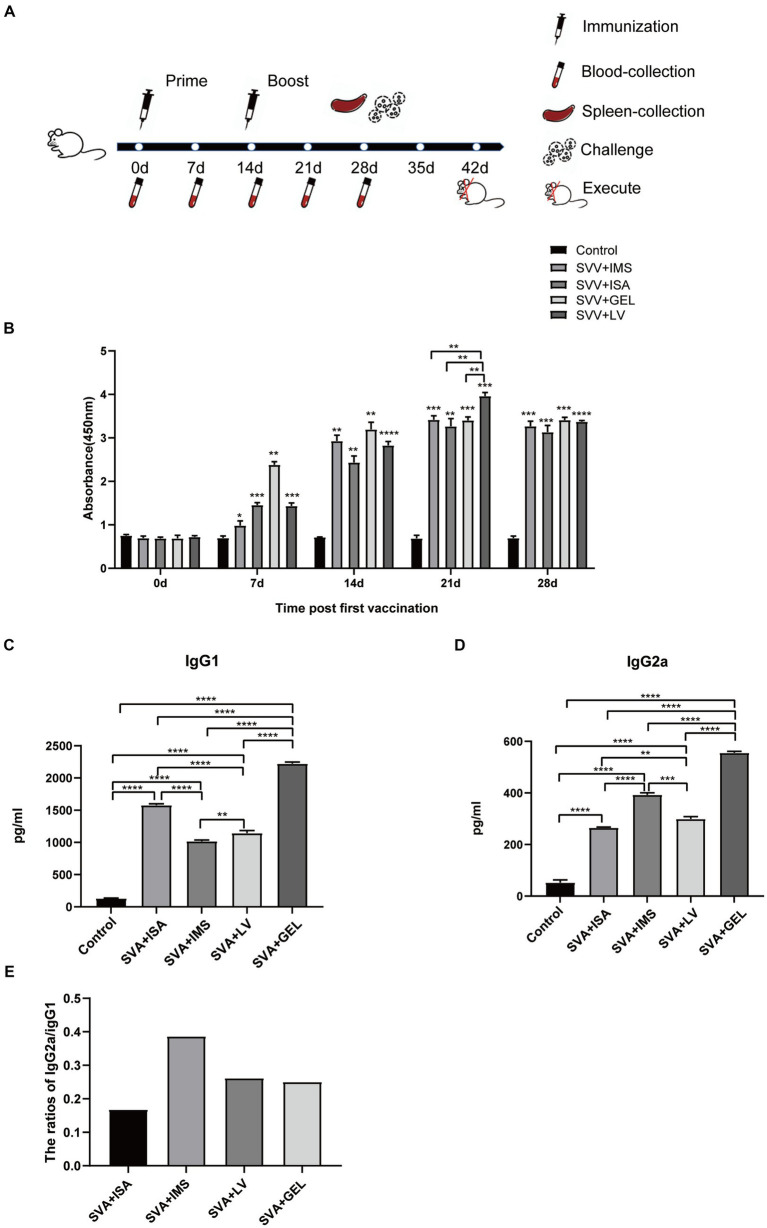
Immunogen-specific antibody levels in mice immunized with different adjuvanted vaccines. **(A)** Immunization schedule and sampling time. **(B)** Levels of immunogen-specific antibodies in sera collected from mice at 0, 7, 14, 21, and 28 d post-immunization as measured by indirect ELISA. **(C)** Levels of IgG1 subtype antibodies in sera of mice collected on d 7 after booster immunization. **(D)** IgG2a subtype antibody levels in sera of mice collected on d 7 after booster immunization. Results expressed as mean ± standard deviation (n = 4), as based on absorbance values measured at OD_450nm_. ^*^p < 0.05, ^**^p < 0.01, ^***^p < 0.001, ^****^p < 0.0001. **(E)** Results of antibody subtype analysis.

To investigate the immunogenic effects of inactivated vaccines formulated with different adjuvants, we administered two subcutaneous multipoint injections to mice over the course of a 28-d immunization cycle. Blood samples were collected from retro-orbital veins at 0, 7, 14, 21, and 28 d following the initial immunization then sera obtained from these samples were aliquoted and stored at −80°C for future use (Figure 4A). Subsequently, measurements of SVA-specific antibody levels in sera collected 0, 7, 14, 21, and 28 d after the initial immunization revealed that sera of all four vaccine-immunized groups contained SVA-specific antibodies, with levels of SVA-specific antibodies peaking at 21 d after the initial immunization. Furthermore, serum SVA-specific antibody levels of the SVA-LV group were higher than corresponding levels of the other three groups (^**^p < 0.01), while no SVA-specific antibodies were detected in control group sera (Figure 4B).

In mice, Th1-associated responses favor IgG2a antibody production, while Th2-associated responses lead to IgG1 production. After primary immunization, mouse sera obtained 21 d later exhibited significantly higher levels of both IgG1 and IgG2a antibodies in all four immunized groups as compared to corresponding control group levels (^****^p < 0.0001). Moreover, the SVA-GEL group exhibited higher levels of IgG1 and IgG2a antibodies than the other three vaccine-immunized groups (^****^p < 0.0001) (Figures 4C,D). Notably, the IgG2a/IgG1 ratios of all four vaccine-immunized groups were < 1. Taken together, these results suggest that all four vaccines elicited mixed Th1 and Th2 immune responses that were skewed towards Th2-type responses (Figure 4E).

### 3.5 Detection of cytokines after immunization of mice

**Figure 5:**
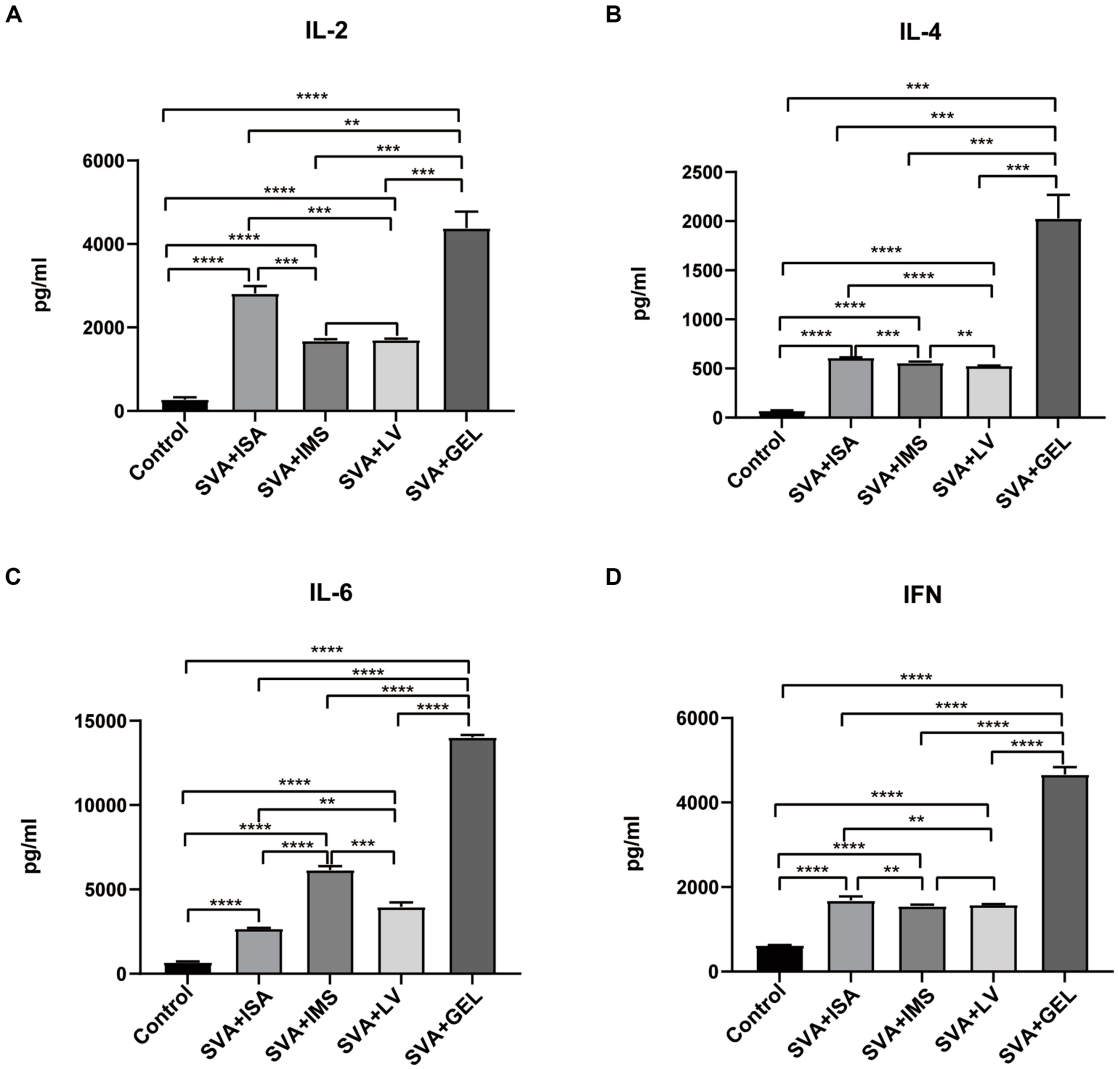
Serum cytokine levels measured post-immunization, with results expressed as the mean ± standard deviation (*n* = 4) of absorbance values measured at OD_450nm_. **(A)** IL-2 levels. **(B)** IL-4 levels. **(C)** IL-6 levels. **(D)** IFN levels. ^**^p < 0.01, ^***^p < 0.001, ^****^p < 0.0001.

To further evaluate effects of vaccination on mouse serum levels of IL-2, IL-4, IL-6, and IFN, sera collected 7 d after booster immunizations were measured using ELISAs. The results demonstrated that levels of the abovementioned cytokines in sera of mice of all four immunized groups were significantly higher than corresponding levels of the control group (^****^p < 0.0001), while cytokine levels of the SVA-GEL group were significantly higher than corresponding levels of the other three immunized groups (^**^p < 0.01, ^***^p < 0.001, ^****^p < 0.0001 for SVA-ISA, SVA-IMS, SVA-LV, respectively) (Figures 5A–D). Additionally, changes in cytokine levels were consistent with observed changes in IgG antibody subtype profiles. Taken together, the immunogenicity or the SVA-GEL vaccine was superior to that of each of the other three vaccines.

### 3.6 Post-immunization neutralizing antibody levels and splenic lymphocyte proliferation responses

**Figure 6:**
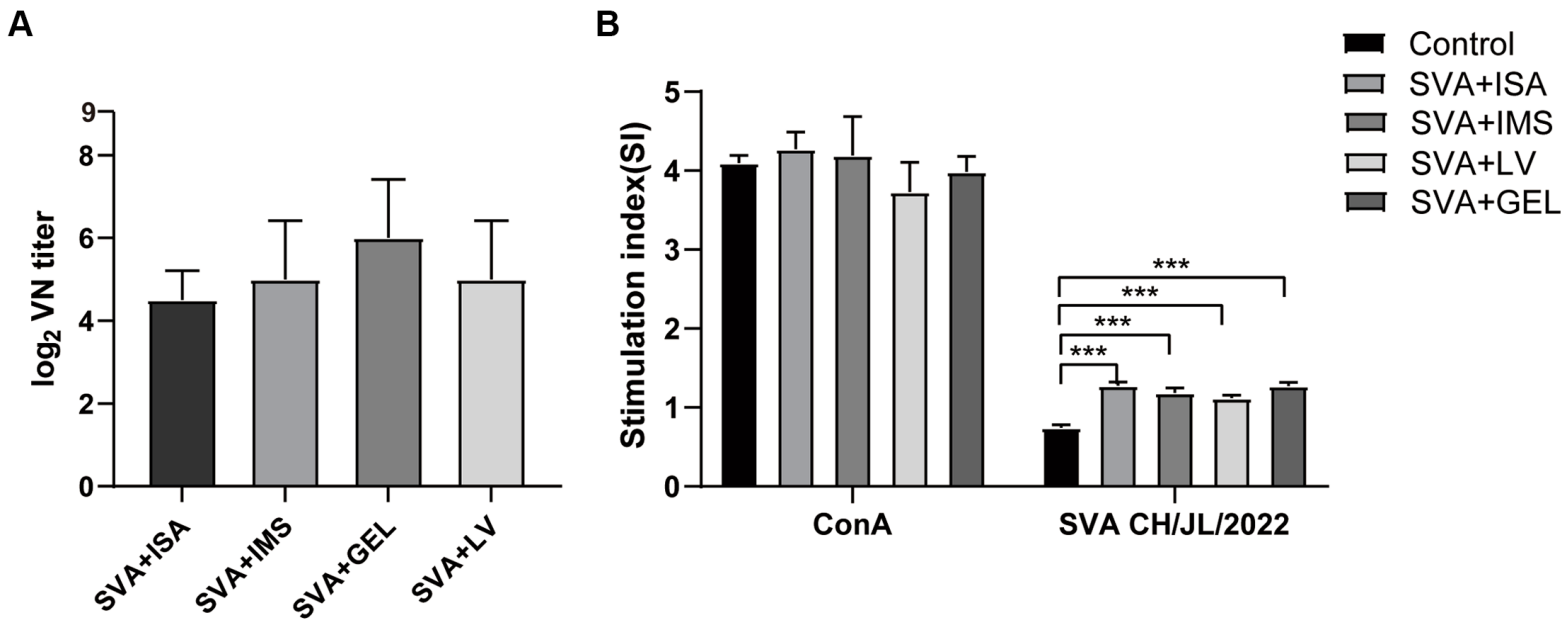
Results of post-immunization serum neutralizing antibody assays and splenic lymphocyte proliferation assays. **(A)** Potencies of neutralizing antibodies in sera collected 7 d after vaccine boosting of immunized mice. **(B)** Splenic lymphocyte proliferation responses of mice in each immunized group as determined using the CCK-8 method, with results expressed as the stimulation index (SI). SI is calculated using the formula SI=OD_experimental group_-OD_background_/OD_negative control group_-OD_background_. Data for each group are expressed as mean ± standard deviation (*n* = 3), ^***^p < 0.001.

To verify the presence of neutralizing antibodies in the sera of the four immunized groups of mice, we collected serum samples 7 d after the mice received booster immunizations. These samples were then inactivated and pipetted into wells of 96-well plates containing both cultured BHK-21 cells and SVA CH/JL/2022 for the purpose of determining serum neutralizing antibody titers based on serum blocking of SVA infection of cells. The results revealed that mice in all four immunization groups produced neutralizing antibodies. Serum titers of neutralizing antibody of mice in the SVA-ISA immunization group ranged from 1:16 to 1:32, while corresponding titers of the SVA-IMS group ranged from 1:16 to 1:64, of the SVA-GEL group ranged from 1:32 to 1:128, and those of the SVA-LV group ranged from 1:16 to 1:64 (Figure 6A).

On the 28th day after the initial immunization, spleens of mice in each group were harvested and splenic lymphocyte proliferation was assessed using the CCK-8 method. The results presented in Figure 6B show no significant difference between SI values of lymphocytes stimulated non-specifically with the mitogen concanavalin A (ConA) and SI values obtained for the four immunized groups (*p* > 0.05), with SI values of all of these groups significantly exceeding that of the control group (*p* < 0.001). The results of the abovementioned experiments showed that all four vaccines were able to induce strong lymphocyte proliferation in mice, while the potency of neutralizing antibody elicited by SVA-GEL immunization exceeded that of each of the other three vaccinated groups.

### 3.7 Histopathologic analysis after challenge with infectious SVA

**Figure 7:**
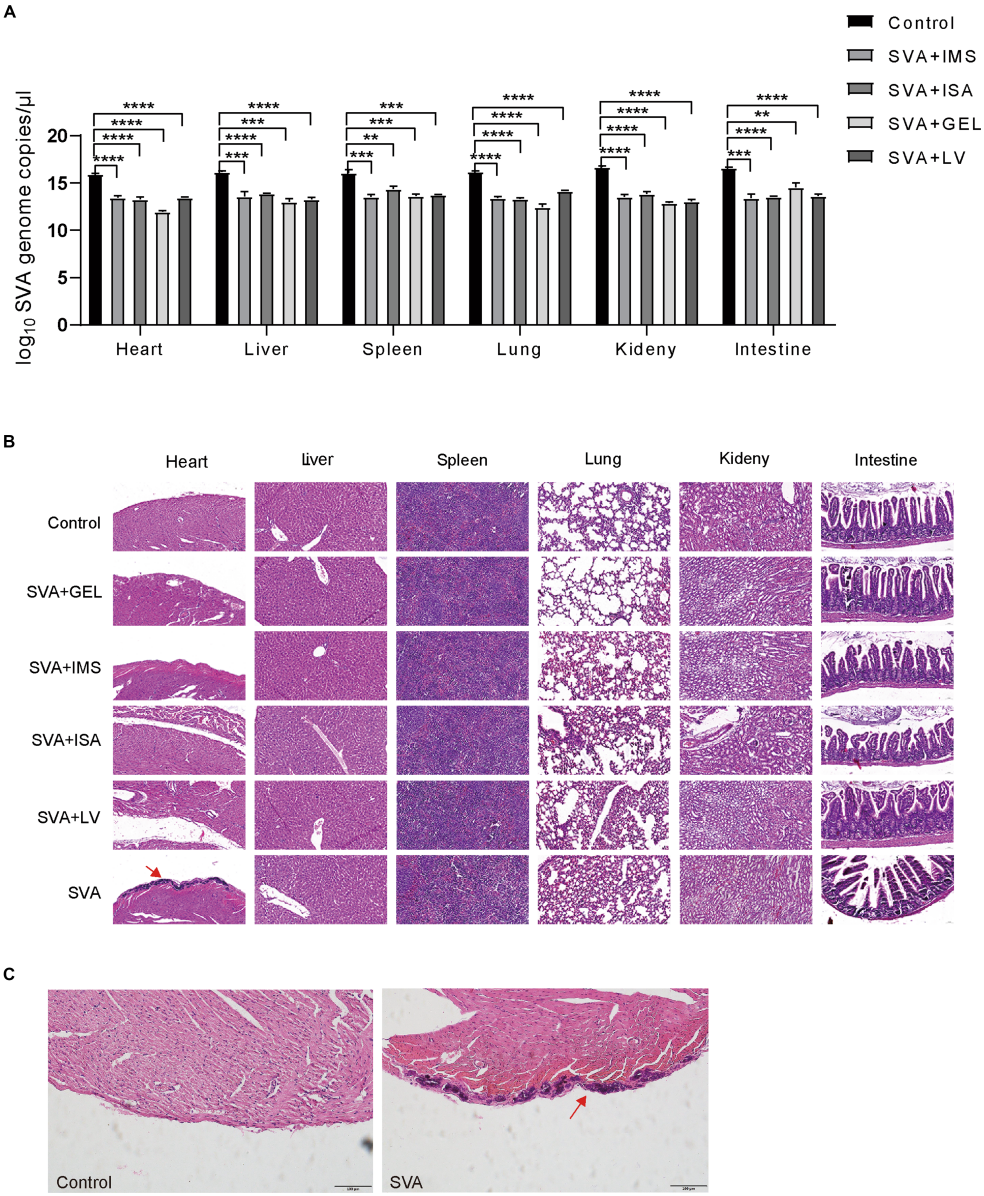
Protection of mice against challenge with infectious SVA at 28 d post-immunization. **(A)** Relative SVA levels in heart, liver, spleen, lung, kidney, and duodenal tissues of mice on day 14 after SVA challenge via inoculation. **(B)** Pathologic examination of mouse hearts after SVA inoculation. A: SVA-PBS; B: SVA-ISA; C: SVA-IMS; D: PBS; E: SVA-GEL; F: SVA-LV. Magnification 40×. **(C)** Light microscopic appearance of the heart of the SVA-infected mice or mice in control group (von Kossa stain, original magnification ×40).

On day 14 after challenge with viable SVA CH/JL/2022 via inoculation, mice were euthanized via cervical dislocation then autopsied to assess effects of vaccination on viral loads in their organs. The results demonstrated significantly lower SVA CH/JL/2022 levels in heart, liver, spleen, lung, kidney, and duodenum, as compared to corresponding levels in organs of unimmunized mice (Figure 7A). Furthermore, most organs of mice in both vaccinated and unvaccinated groups showed no significant pathological signs (Figure 7B). However, dissected hearts of all mice in the unimmunized group exhibited distinct white fibrous streaks. After the hearts sections were stained with HE or Von Kossa, and viewed under an optical microscope, hearts of the unimmunized group were found to exhibit histological signs of severe pericardial calcification, whereas mice in the immunized group showed no histological signs of heart damage (Figures 7B,C). Taken together, these results demonstrated that SVA-ISA, SVA-IMS, SVA-GEL, and SVA-LV vaccines can protect mice from SVA infection.

## 4 Discussion

As of December 2023, SVA outbreaks had affected over half of Chinese provinces, autonomous regions, and municipalities. Phylogenetic analysis of Chinese SVA isolates obtained from these outbreaks revealed they could be classified into two clusters and five genetically distinct branches (11, 25). Some Chinese strains showed evidence of genetic mutation, particularly those present in specimens obtained from asymptomatic SVA-infected pigs (13). In this study a new strain of SVA, designated SVA CH/JL/2022, was isolated for the first time from pathological samples obtained from SVA-infected pigs residing on a farm in Jilin Province, China. Based on results of analyses of whole-genome sequences of SVA CH/JL/2022 and other SVA strains and the phylogenetic tree constructed from these sequences, we speculate that SVA CH/JL/2022 strain was evolutionarily related to the other Chinese isolates and formed an evolutionary branch. In addition, SVA CH/JL/2022 strain shared the greatest homology with that of the first SVA strain, SVV-001 (26). These results indicate that the nucleotide mutations of the epidemic strain were continuing to evolve in China.

Of note, it has been reported that SVA has been detected in mice living on pig farms and successfully isolated from mouse tissues (3, 8). As previously reported, the viral RNA or histopathological damages could be detected in the heart, liver, spleen, lung, kidney, intestine and brain tissues of the immunized mice (8). Interestingly, infection of mice with the SVA CH/JL/2022 strain was observed to specifically target heart tissues, leading to severe pericardial calcification, while no histopathological damages were observed in other corresponding tissues of SVA CH/JL/2022-infected mice, as was not previously reported (8). Additionally, an analysis of whole-genome sequences revealed numerous genomic nucleotide mutations associated with SVA CH/JL/2022 strain that would lead to amino acid substitutions. These findings suggest that SVA is rapidly evolving into potentially more phenotypically virulent subtypes that may cause future pandemics. However, further research is needed to better understand the roles of SVA polyprotein amino acid substitutions on SVA pathogenicity and immunogenicity.

While various vaccine development platforms have been employed to create inactivated vaccines to combat the current PIVD pandemic, no commercial vaccines are yet available to prevent and control SVA-induced PIVD. The lack of progress in vaccine development may be attributed to the high diversity of SVA isolates, their rapid evolution, high costs associated with vaccine evaluation in pigs, and the lack of suitable animal models of SVA infection. Recently, The study has proven that mouse was a candidate animal model for the primary evaluation of the immunogenicity and protection efficacy of SVA vaccines (8). Thus, in this study a mouse model was used to evaluate the immunogenicity and protective efficacy of four inactivated SVA vaccines after the SVA CH/JL/2022 immunogen was confirmed to proliferate in BHK-21 cells and infect mice. And the results we obtained using a mouse model demonstrated that this SVA strain may serve as an effective immunogenic strain that can be propagated in cell lines *in vitro* and evaluated for *in vivo* efficacy in mice.

Considerable efforts have been made over the past 10 years to develop vaccines against SVA-induced PIVD, and the main studied vaccine types include inactivated vaccines (8, 14, 17). To solve the global problem caused by SVA infection, more vaccines need to be developed. Adjuvants are critical components of subunit and certain types of inactivated vaccines that enable such vaccines to elicit more robust and longer lasting specific immune responses (27, 28). In this study, we evaluated four commercial adjuvants (GEL 02, ISA 201, IMS1313, and LV) for use in our inactivated SVA vaccines. Indeed, the adjuvants of ISA 201 and IMS1313 were mixed with inactivated SVA had induced humoral and cellular immunity obviously, respectively (8, 14, 17). In addition, Liu et al. have investigated an SVA-inactivated vaccine based on screening of adjuvants of ISA 201 (SVA-ISA) and IMS 1313 (SVA-IMS) and the results showed that the inactivated vaccine, SVA-IMS, had better immunoreactivity and protection efficacy than SVA-ISA, and both the SVA-IMS and SVA-ISA combinations could resist challenge from a virulent SVA strain (16). Recently, some studies revealed that the Montanide GEL 01 ST, a novel water-based adjuvant, can effectively improve the protective effect of vaccines with few or no side effects in mice and pigs (29–31), and the adjuvant Montanide GEL 02 PR is a new water-based adjuvant updated from the Montanide GEL 01 ST (31), however, there are no reports of this adjuvant being tested in SVA inactivated vaccines.

The immunogen component of the four vaccines was prepared by inactivating 10^6.375^ TCID_50_/ml of SVA CH/JL/2022 with formaldehyde (4‰ final concentration), which is the most commonly used inactivator in currently approved viral vaccines (31). The inactivated SVA immunogen was then mixed with each of the four adjuvants according to the instructions provided with the adjuvants to generate four different vaccines. The resulting vaccines were evaluated for immunogenicity and protective efficacy against SVA infection in mice receiving dorsal subcutaneous multipoint vaccine injections prior to challenge with infectious SVA via inoculation. Our results revealed that all four vaccines effectively stimulated mouse splenic lymphocyte proliferation, promoted production of both Th1 and Th2 cytokines, and elicited predominantly Th2-type immune responses. Furthermore, viral RNA levels in organs of mice immunized with SVA-ISA, SVA-IMS, SVA-GEL, and SVA-LV vaccines were significantly reduced and no pathogenic organ abnormalities observed. These findings demonstrate that all four vaccines effectively protected mice from SVA infection.

Notably, the SVA-LV group exhibited a higher serum level of SVA-specific antibodies at day 14 after booster administration than the other vaccinated groups. However, it is important to mention that the SVA-LV vaccine did not provide greater safety and immunogenicity as compared to the other vaccines when administered *in vivo* to mice (data not shown). In contrast, as compared to the other vaccines, the SVA-GEL vaccine exhibited greater *in vivo* immunogenic potency, greater protection from SVA CH/JL/2022 infection (Figures 4–6), and did not trigger adverse reactions (data not shown). Nevertheless, further clinical trials are needed to confirm whether this vaccine formulation can provide cross-protection against diverse, heterologous SVA field strains.

## 5 Conclusion

Here we report genetic and pathogenic characteristics of a novel SVA strain derived from an isolate obtained from a pig in Jilin Province, China. This strain was then used to generate four inactivated SVA vaccines containing commercial adjuvants GEL, ISA 201, IMS1, or LV. Comparisons of vaccine efficacies and performance indicators using a mouse model of SVA infection demonstrated that all four adjuvanted vaccines containing the inactivated SVA CH/JL/2022 immunogen exhibited good immunogenicity and effectively protected immunized mice from SVA infection. Ultimately, the inactivated SVA-GEL vaccine developed in this study provided superior efficacy as compared to the other three vaccine candidates and thus may serve as an effective tool for preventing SVA infection.

## Data availability statement

The datasets presented in this study can be found in online repositories. The names of the repository/repositories and accession number(s) can be found at: https://www.ncbi.nlm.nih.gov/genbank/, OP562896.

## Ethics statement

The animal study was approved by the Institutional Animal Care and Use Committee of Jilin University. The study was conducted in accordance with the local legislation and institutional requirements.

## Author contributions

BW: Data curation, Investigation, Methodology, Validation, Writing – original draft. FeiG: Supervision, Writing – review & editing, Funding acquisition, Validation. RH: Data curation, Software, Writing – review & editing. HH: Data curation, Resources, Writing – review & editing. GW: Resources, Software, Writing – review & editing. ZC: Data curation, Writing – review & editing. YZ: Data curation, Writing – review & editing. HL: Investigation, Resources, Writing – review & editing. DS: Supervision, Writing – review & editing. FengG: Supervision, Writing – review & editing. WH: Data curation, Supervision, Writing – review & editing. YL: Conceptualization, Supervision, Validation, Writing – original draft, Writing – review & editing.

## Footnotes

^1^https://www.ncbi.nim.nih.gov/genbank
